# TAVI plus PCI versus SAVR plus CABG: Long-term outcome of a multicentre-registry

**DOI:** 10.1007/s00392-025-02755-9

**Published:** 2025-09-11

**Authors:** A. Stundl, L. Preuss, A. Prinzing, J. C. Voran, H. Seoudy, I. Mesanovic, V. Obermeier, G. Lutter, M. Potratz, G. Buglio, A. Pohlmeyer, R. Thalmann, P. Hoppmann, C. Bradaric, H. Ruge, M. Erlebach, R. Lange, K. L. Laugwitz, T. Pühler, T. Rudolph, S. Bleiziffer, M. Krane, D. Frank, C. Kupatt

**Affiliations:** 1 Department of Internal Medicine I, Cardiology, Klinikum Rechts Der Isar, TUM School of Medicine & Health, Technische Universität München, Munich, Germany; 2 German Centre for Cardiovascular Research (DZHK), Partner Site Munich, Munich, Germany; 3 Department of Cardiovascular Surgery, German Heart Center Munich, TUM School of Medicine & Health, Technische Universität München, Munich, Germany; 4 Department of Internal Medicine III, Cardiology and Critical Care, University Hospital Schleswig-Holstein, Campus Kiel, Kiel, Germany; 5 German Centre for Cardiovascular Research (DZHK), Partner Site Hamburg/Kiel/Lübeck, Kiel, Germany; 6 Department of Cardiovascular Surgery, University Hospital Schleswig-Holstein, Campus Kiel, Kiel, Germany; 7 Department of General and Interventional Cardiology and Angiology, Herz- Und Diabetes-Zentrum, Bad Oeynhausen, Germany; 8 Department of Cardiothoracic Surgery, Herz- Und Diabetes-Zentrum, Bad Oeynhausen, Germany

**Keywords:** Aortic stenosis, Coronary artery disease, Intermediate risk, Transcatheter aortic valve implantation, Surgical aortic valve replacement

## Abstract

**Background:**

In elderly patients with severe aortic stenosis (AS), concomitant coronary artery disease (CAD) is common. Escpecially for intermediate-risk patients eligible for both interventional and surgical treatment, long-term benefit of either approach remains unclear.

**Objectives:**

To compare long-term outcomes of TAVI + PCI versus SAVR + CABG in intermediate-risk AS-CAD patients (logistic EuroSCORE 10–20%, EuroSCORE II 4–9%).

**Methods:**

This retrospective multicentre study included 366 patients treated between 2012 and 2020: 211 underwent TAVI + PCI and 155 received SAVR + CABG. The primary endpoint was all-cause mortality up to three years; secondary outcomes followed VARC-3 criteria.

**Results:**

Mortality rates were similar at 30 days (4.8% vs. 8.4%, p = 0.16), six months (12.4% each), one year (18.1% vs. 15.7%) and two years (24.9% vs. 20.1%). At three years, mortality was higher after TAVI + PCI (37.1% vs. 25.5%, *p* = 0.02), though CAD complexity was greater in the SAVR + CABG group (SYNTAX Score 22.2 vs. 15.9, *p* < 0.001). TAVI + PCI patients were older (81.1 vs. 78.5 years, *p* < 0.001), but surgical risk was comparable (EuroSCORE II 6.4% vs. 6.2%). Surgical patients experienced more complications, including delirium, stroke, acute kidney injury, major bleedings and transfusion needs. After propensity score matching (154 patients per group), 3-year mortality no longer differed significantly (33.8% vs. 25.9%, p = 0.14).

**Conclusion:**

Both TAVI + PCI and SAVR + CABG yield comparable long-term outcomes in intermediate-risk AS-CAD patients. Although early complications were more common with surgery, there was a trend towards improved long-term survival.

**Graphical Abstract:**

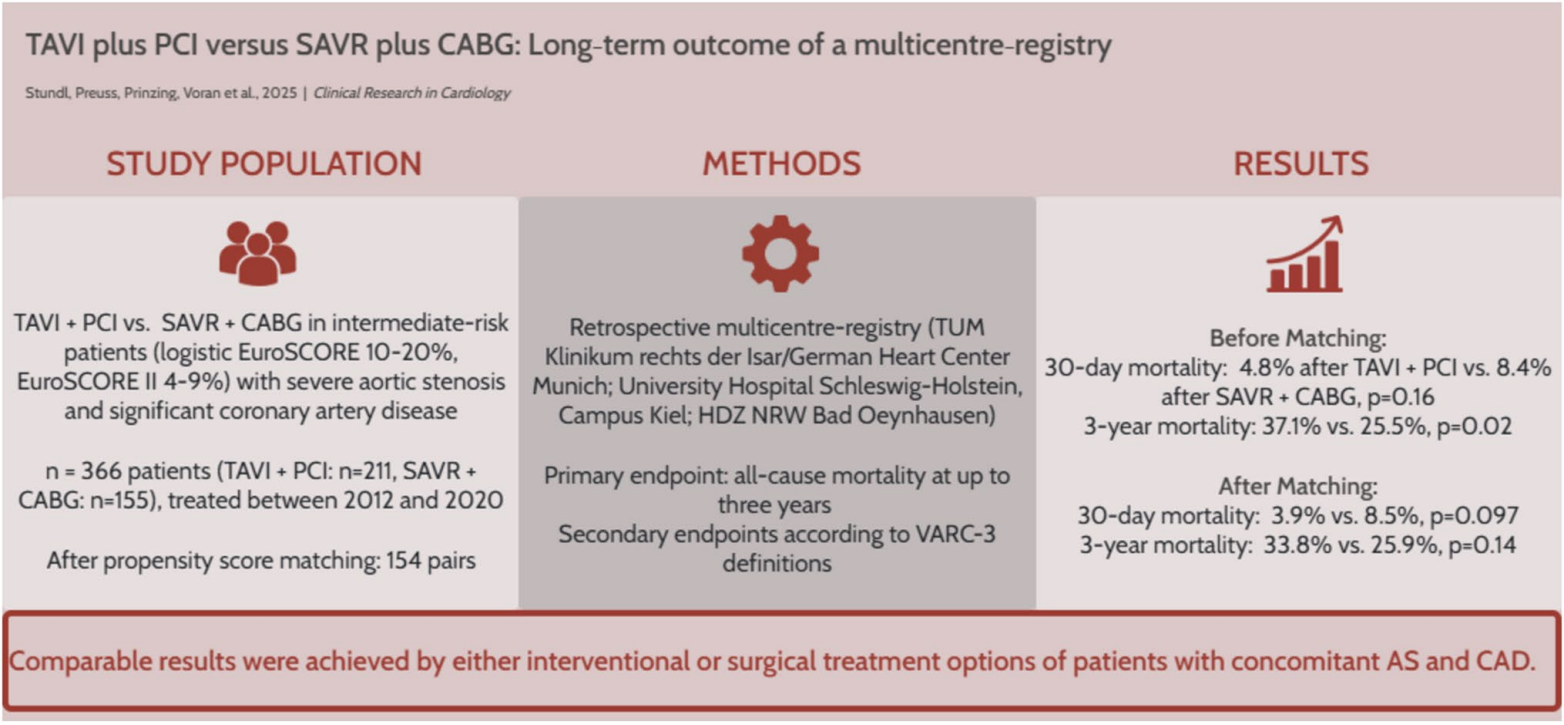

**Supplementary Information:**

The online version contains supplementary material available at 10.1007/s00392-025-02755-9.

## Introduction

The prevalence of severe aortic stenosis (AS) ranges from 2.4% to 3.4% in patients older than 75 years, rendering AS the most commonly acquired valvular disease leading to replacement in the western world [1–3]. Multiple randomised controlled trials demonstrated that transcatheter aortic valve implantation (TAVI) does not perform inferior to surgical aortic valve replacement (SAVR) in elderly patients, across increased or high [4, 5], intermediate [6, 7] and low operative risk levels [8]. Therefore, current guidelines of the ESC/EACTS recommend use of TAVI in older patients (≥ 75 years), or in those who are high-risk (STS-PROM/EuroSCORE II > 8%) or unsuitable for surgery [9]. Vice versa, SAVR is recommended in younger patients who are at low risk for surgery (< 75 years and STS- PROM/EuroSCORE II < 4%) or in patients who are operable and unsuitable for transfemoral TAVI [9].

One of the most frequent comorbidities of AS is coronary artery disease (CAD), as the prevalence of both AS and CAD increases with age [1]. Accordingly, CAD occurs in 45 to 75% of patients in TAVI registries [10–13]. Moreover, the combination of AS and CAD carries a higher risk for adverse events, even if both entities were treated separately. ESC/EACTS [14] and ACC/AHA [15] guidelines recommend treatment with SAVR and CABG for surgical patients in a combined procedure, without discerning risk stratae or complexity of the CAD. For the management of CAD in TAVI patients, there are no established recommendations to this day due to scarce long-term data.

Here, we analyse a cohort of intermediate-risk patients from three German high-volume heart centers providing both surgical and interventional treatment options (German Heart Center Munich, University Hospital Schleswig–Holstein, Campus Kiel, and HDZ NRW Bad Oeynhausen). The outcomes after either interventional or surgical treatment were compared during a follow-up period of three years.

## Methods

This was a retrospective multicentre study including patients with severe AS and significant CAD who underwent either TAVI + PCI or SAVR + CABG between 2012 and 2020 in one of the three participating heart centres. Data regarding medical history, procedural details and clinical outcomes were recorded in a database. The decision to perform either surgery or percutaneous intervention was taken individually by the Heart Team at each centre. The study was conducted in accordance with the ethical standards stated in the Declaration of Helsinki. All institutional ethics committees approved the study and written consent to the procedures (TAVI + PCI or SAVR + CABG) and to data registration was obtained from every included patient. Follow-up was completed using in-hospital medical records and conducting telephone interviews.

### Patient population

A total of 366 patients undergoing either treatment with TAVI + PCI or SAVR + CABG were included according to our inclusion criteria. These encompassed age > 18 years, severe AS (defined by an aortic valve area ≤ 1 cm^2^), significant CAD (defined as stenosis > 50% in the coronary angiography) and intermediate risk (defined by logistic EuroSCORE 10–20% and EuroSCORE II 4–8%). Interventional treatment was performed as staged procedures with a median interval of 30 days between PCI and TAVI (IQR 6.0–42.0), while surgical treatment consisted of a single operation (SAVR + CABG).

### Study endpoints

The primary endpoint was all-cause mortality after 30, 180, 365, 730 and 1095 days. As secondary endpoints, outcomes according to Valve Academic Research Consortium-3 criteria occurring during the first 30 days after valve treatment were recorded (all stroke, myocardial infarction, need for pacemaker implantation, minor and major vascular complications, major bleedings, need for transfusions, acute kidney injury).

### Statistical analysis

Continuous variables are expressed as mean ± standard deviation (SD) or median (interquartile range) and categorical variables as absolute numbers and percentages. Comparisons were performed using t-test for normally distributed continuous variables, while the Mann–Whitney U test was used for continuous variables with skewed distribution. The Chi-square test was used to compare categorical variables. Given the significant differences in epidemiologic data with possible prognostic relevance, we applied propensity score matching to the interventional and surgical treatment arms.

A propensity score (PS) was calculated for each patient to estimate the propensity for being included in one of the two treatment groups (TAVI + PCI vs. SAVR + CABG). This was performed by using a multivariate logistic regression. Covariates that were included in the calculation of the PS were age, sex, BMI, STS-PROM score, diabetes mellitus, arterial hypertension, left ventricular ejection fraction (LVEF), main stem stenosis, atrial fibrillation, pulmonary hypertension, NYHA class, chronic obstructive pulmonary disease (COPD), peripheral arterial disease (PAD), cerebrovascular disease (CVD), previous myocardial infarction and previous heart surgery. A 1-to-1 nearest neighbor matching algorithm was used to identify PS-matched pairs. Resulting were 154 patients in each group (308 in total) with an adequately balanced covariate distribution. Kaplan–Meier estimates and the log-rank test were used to compare and graphically display the mortality rates. P-values < 0.05 were considered statistically significant for all statistical tests. The analyses were performed using SPSS version 29 and R version 4.4.1 (2024–06-14 ucrt), packages “tidyverse” and “MatchIt”.

## Results

### Baseline characteristics

As depicted in Table 1, epidemiological data indicate an elderly study cohort with a significant difference in mean age (81.1 ± 5.7 vs. 78.5 ± 5.0 yrs., p < 0.001). The EuroSCORE II indicated similar surgical risk for open heart surgery (6.4 ± 3.1 vs. 6.2 ± 2.9%, p = 0.53), while the STS-PROM was higher in the interventional group (4.9 ± 2.5 vs. 4.2 ± 2.1, p = 0.007). Regarding individual cardiovascular risk factors, however, there were no significant differences except for a higher BMI in surgical patients (27.7 ± 4.7 vs. 26.7 ± 4, p = 0.002). Concerning cardiovascular comorbidities (see Table 2), pulmonary hypertension was more prevalent in the TAVI + PCI group (43.1% vs. 28.4%, p = 0.004), while the incidences of atrial fibrillation, extracardiac arteriopathy, previous stroke and myocardial infarction were similar. Remarkably, the majority of patients in the SAVR + CABG group presented with 3-vessel-CAD (51.6% vs. 41.2% in the TAVI + PCI group, p = 0.049). Also pointing towards a more complex CAD pattern, main stem stenosis was more frequent in surgical patients (29.7% vs. 17.1%, p = 0.004) and they had a significantly higher SYNTAX Score I (22.2 ± 13.4 vs. 15.9 ± 10.7, p < 0.001).

**Table 1:** Epidemiology and comorbidities in the unmatched population

	TAVI + PCI (*n* = 211)	SAVR + CABG (*n* = 155)	*p* value
Age (years)	81.1 ± 5.7	78.5 ± 5.0	** < 0.001**
Male Sex, n (%)	124 (58.8)	87 (56.1)	0.796
Logistic EuroSCORE (%)	14.3 ± 2.8	13.8 ± 2.7	0.061
EuroSCORE II (%)	6.4 ± 3.1	6.2 ± 2.9	0.523
STS-PROM (%)	4.9 ± 2.5	4.2 ± 2.1	**0.007**
BMI (kg/m^2^)	26.7 ± 4.7	27.7 ± 4.7	**0.002**
Obesity, n (%)	46 (21.8)	40 (25.8)	0.354
Diabetes mellitus, n (%)	63 (30.0)	51 (32.9)	0.723
- dietary, n (%)	11 (5.2)	6 (3.9)	
- oral antidiabetics, n (%)	22 (10.4)	23 14.8)	
- insulin-dependent, n (%)	30 (14.2)	22 (14.2)	
Hypertension, n (%)	198 (93.8)	142 (91.6)	0.413
Hyperlipidaemia, n (%)	178 (84.4)	127 (81.9)	0.539
Nicotine abuse, n (%)	40 (28.2)	40 (42.6)	0.066
** -** history of smoking, n (%)	19 (9.0)	17 11.0)	
** -** current smoking, n (%)	41 (19.4)	28 (18.1)	
Dialysis, n (%)	8 (3.8)	2 (1.3)	0.147
COPD, n (%)	42 (19.9)	22 (14.2)	0.149

Categorial variables are presented as number and percentegas, continuous variables as mean ± standard deviation. *STS – PROM* Society of thoracic surgeons – predicted risk of operative mortality, *BMI* body mass index, *COPD* chronic obstructive pulmonary disease, *TAVI* transcatheter aortic valve implantation, *PCI* percutaneous coronary intervention, *SAVR* surgical aortic valve replacement, *CABG* coronary artery bypass grafting

**Table 2:** Cardiovascular status in the unmatched population

	TAVI + PCI (*n* = 211)	SAVR + CABG (*n* = 155)	*p* value
Atrial fibrillation, n (%)	83 (39.3)	48 (31.0)	0.099
NYHA IV, n (%)	21 (10.0)	10 (6.5)	0.235
Extracardial atheropathy, n (%)	80 (37.9)	54 (34.8)	0.546
CVD, n (%)	60 (28.4)	37 (23.9)	0.328
PAD, n (%)	39 (18.5)	26 (16.8)	0.672
Pulmonary hypertension, n (%)	91 (43.1)	44 (28.4)	**0.004**
Previous stroke, n (%)	25 (11.8)	13 (8.4)	0.283
Previous myocardial infarction, n (%)	50 (23.7)	37 (23.9)	0.969
Previous cardiac surgery, n (%)	22 (10.4)	8 (5.2)	0.162
LVEF (%)	52.0 ± 10.7	50.9 ± 13.2	0.394
Aortic valve area (cm^2^)	0.76 ± 0.16	0.79 ± 0.25	0.213
Peak pressure gradient (mmHg)	64.8 ± 22.8	61.6 ± 23.5	0.271
Mean pressure gradient (mmHg)	40.5 ± 15.0	37.6 ± 17.0	0.116
Coronary artery disease, n (%)	211 (100.0)	155 (100.0)	1.000
- 1-CAD, n (%)	48 (22.7)	28 (18.1)	0.265
- 2-CAD, n (%)	73 (34.6)	47 (30.3)	0.389
- 3-CAD, n (%)	87 (41.2)	80 (51.6)	**0.049**
- main stem stenosis	36 (17.1)	46 (29.7)	**0.004**
SYNTAX Score I (%)	15.9 ± 10.7	22.2 ± 13.4	** < 0.001**
- low SYNTAX Score I (≤ 22), n (%)	74 (80.4)	51 (55.4)	** < 0.001**
- intermediate SYNTAX Score I (23–32), n (%)	11 (12.0)	22 (23.9)	**0.023**
- high SYNTAX Score I (> 32), n (%)	3 (3.3)	11 (12.0)	**0.020**

Categorial variables are presented as number and percentegas, continuous variables as mean ± standard deviation. *NYHA* New York Heart Association, *CVD* cerebrovascular disease, *PAD* peripheral arterial disease, *LVEF* left-ventricular ejection fraction, *CAD* coronary artery disease, *TAVI* transcatheter aortic valve implantation, *PCI* percutaneous coronary intervention, *SAVR* surgical aortic valve replacement, *CABG* coronary artery bypass grafting

### Procedural characteristics

Procedurally (see Table 3), all surgically treated patients underwent general anaesthesia, as opposed to only 47.9% in the interventional group due to 39.3% receiving analgosedation and 12.8% being treated by local anaesthesia only. Procedural time was significantly longer for surgical treatment (266.0 min, IQR 225.−328.0) compared to the sum of TAVI and PCI (108.0 min, IQR 84.0–128.0; p < 0.001).

**Table 3:** Procedural characteristics in the unmatched population

	TAVI + PCI (*n* = 211)	SAVR + CABG (*n* = 155)	*p* value
Analgosedation, n (%)	83 (39.3)	0 (0.0)	** < 0.001**
Local anesthesia, n (%)	27 (12.8)	0 (0.0)	** < 0.001**
General anesthesia, n (%)	101 (47.9)	155 (100.0)	** < 0.001**
Procedural time (min)	108.0 (84.0–128.0)	266.0 (225.0–328.0)	** < 0.001**
Days between PCI and TAVI	30.0 (6.0–42.0)		

Categorial variables are presented as number and percentegas, continuous variables as mean ± standard deviation. *TAVI* transcatheter aortic valve implantation, *PCI* percutaneous coronary intervention, *SAVR* surgical aortic valve replacement, CABG coronary artery bypass grafting.

### Clinical outcomes

**Fig. 1:**
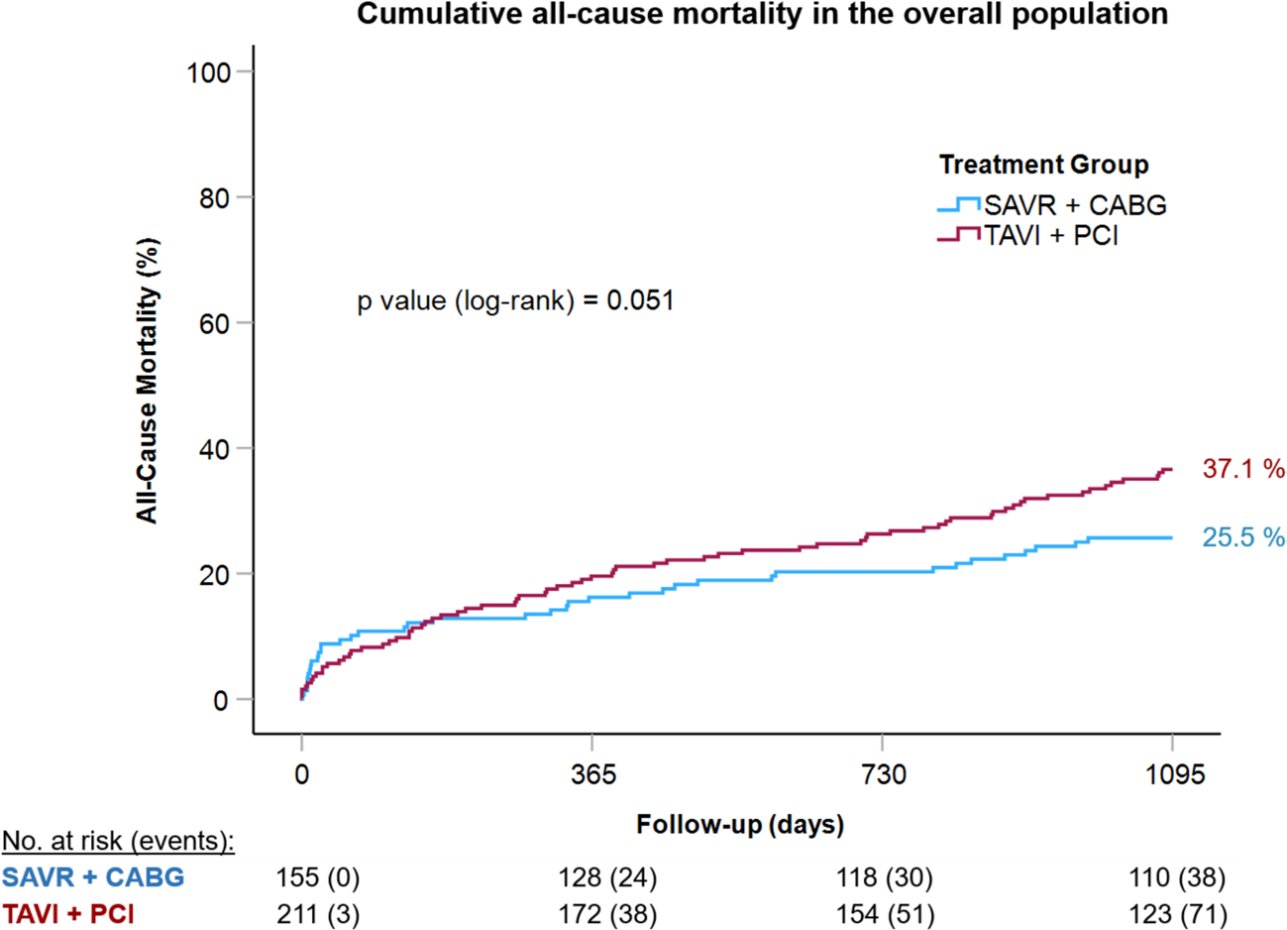
Three-year mortality in the overall population. TAVI: transcatheter aortic valve implantation. PCI: percutaneous coronary intervention. SAVR: surgical aortic valve replacement. CABG: coronary artery bypass grafting

Early postprocedurally, delirium (31.0% vs. 6.2%, p < 0.001) and all stroke (7.7% vs. 1.4%, p = 0.003) were experienced more frequently by the surgical group, as were acute kidney injury (29.0% vs. 11.8%, p < 0.001), major bleedings (36.1% vs. 13.3%, p < 0.001) and the need for transfusions (79.4% vs. 29.4%, p < 0.001). The other postprocedural complications were similarly distributed between both groups, although there was a tendency towards a higher count of minor vascular complications and new pacemaker implantation in the interventional group. Prior to matching, 30-day mortality differed not statistically significantly between the interventional and surgical group (4.8% vs. 8.4%, p = 0.159). While the mortality rates were completely even at 180 days (12.4% respectively), an initial trend towards a higher mortality in the TAVI + PCI group was detected after one year (18.1% vs. 15.7%, p = 0.547), which aggravated at two years (24.9% vs. 20.1%, p = 0.294) and reached statistical significance after three years (37.1% vs. 25.5%, p = 0.022). Detailed results for primary and secondary endpoints are presented in Table 4 and the accompanying Kaplan Meier estimates are shown in Fig. 1.

**Table 4:** Clinical outcomes in the unmatched population

	TAVI + PCI (*n* = 211)	SAVR + CABG (*n* = 155)	*p* value
Delirium, n (%)	13 (6.2)	48 (31.0)	** < 0.001**
Stroke, n (%)	3 (1.4)	12 (7.7)	**0.003**
Myocardial infarction, n (%)	4 (1.9)	2 (1.3)	0.652
Need for transfusions, n (%)	62 (29.4)	123 (79.4)	** < 0.001**
Major bleedings, n (%)	28 (13.3)	56 (36.1)	** < 0.001**
Major vascular complications, n (%)	7 (3.3)	9 (5.8)	0.908
Minor vascular complications, n (%)	23 (10.9)	12 (7.7)	0.310
Acute kidney injury, n (%)	25 (11.8)	45 (29.0)	** < 0.001**
- Grade 1	15 (7.1)	24 15.5)	
- Grade 2	5 (2.4)	7 (4.5)	
- Grade 3	5 (2.4)	14 (9.0)	
Pacemaker implantation, n (%)	22 (10.4)	11 (7.1)	0.272
30-day mortality (%)	10 (4.8)	13 (8.4)	0.159
180-day mortality (%)	26 (12.4)	19 (12.4)	0.991
365-day mortality (%)	38 (18.1)	24 (15.7)	0.547
730-day mortality (%)	51 (24.9)	30 (20.1)	0.294
1095-day mortality (%)	72 (37.1)	38 (25.5)	**0.022**

Categorial variables are presented as number and percentegas, continuous variables as mean ± standard deviation. *TAVI* transcatheter aortic valve implantation, *PCI* percutaneous coronary intervention, *SAVR* surgical aortic valve replacement, *CABG* coronary artery bypass grafting

### Results in the PS-matched cohort

**Fig. 2:**
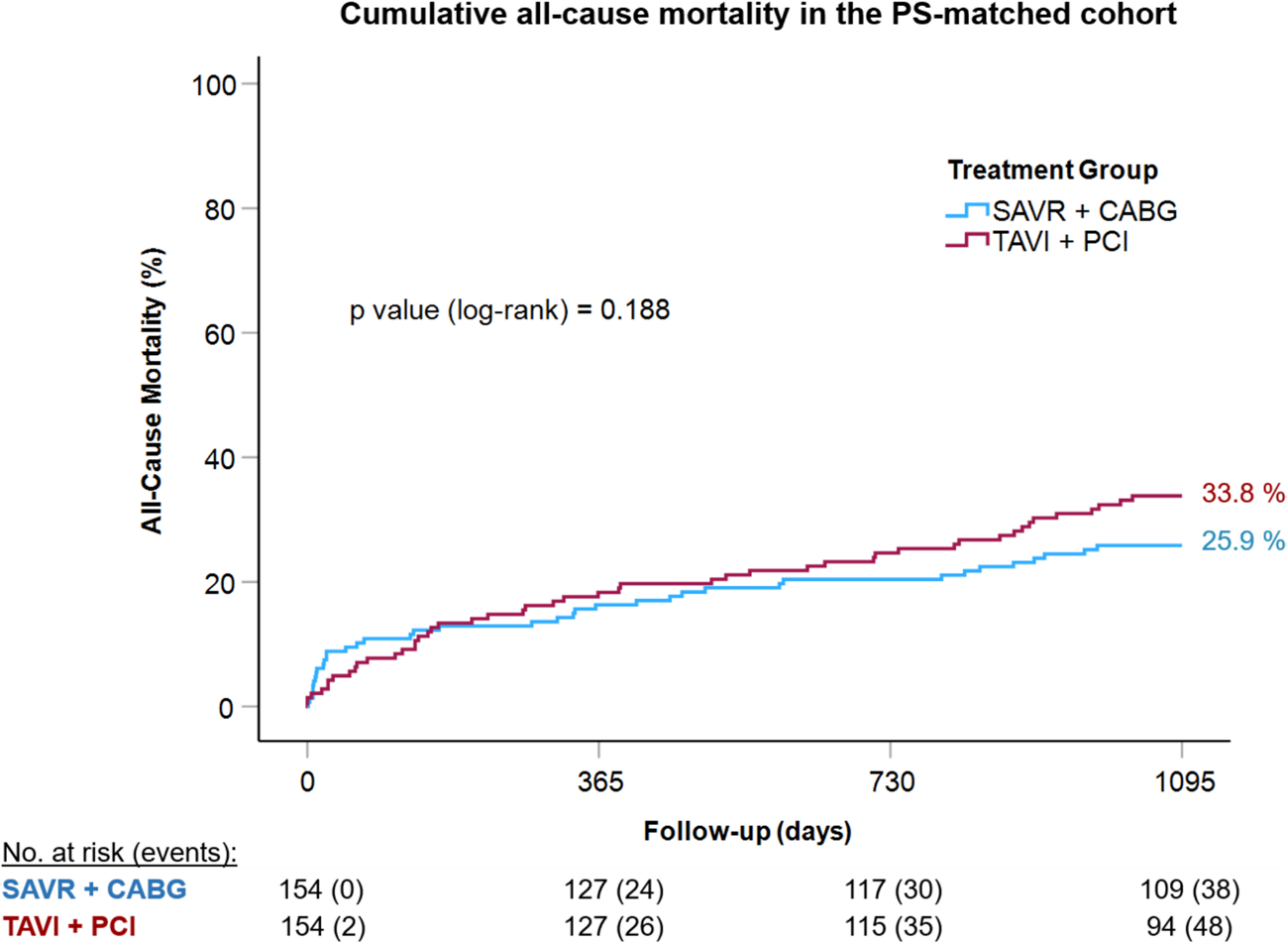
Three-year mortality in the propensity-score matched cohort. TAVI: transcatheter aortic valve implantation. PCI: percutaneous coronary intervention. SAVR: surgical aortic valve replacement. CABG: coronary artery bypass grafting

After propensity score matching, a total of 154 pairs were formed and the risk profiles were well balanced (depicted in Tables 5 and 6). A minor difference persisted for age (79.7 ± 5.2 for TAVI + PCI vs. 78.5 ± 5.0 for SAVR + CABG, p = 0.032), and SYNTAX Score (22.2 ± 13.5 for SAVR + CABG vs. 16.2 ± 10.9 for TAVI + PCI, p < 0.001) (see limitations). Overall, procedural characteristics stayed unchanged (see Table 7), as were the persistingly higher rates of delirium (31.2% vs. 6.5%, p < 0.001), all stroke (7.8% vs. 1.9%, p = 0.017), acute kidney injury (29.2% vs. 12.3%, p = 0.003), major bleedings (36.4% vs. 10.4%, p < 0.001) and requirement for transfusions (79.2% vs. 29.9%, p < 0.001) in the surgical group. However, concerning survival, an important distinction was to be found: At three years, the mortality rates were still numerically higher by 8% (33.8% after TAVI + PCI vs. 25.9% after SAVR + CABG, p = 0.139), but they no longer differed significantly from each other. Matched outcomes are presented in Table 8 and the Kaplan Meier estimates in Fig. 2.

**Table 5:** Epidemiology and comorbidities in the matched cohort

	TAVI + PCI (*n* = 154)	SAVR + CABG (*n* = 154)	*p* value
Age (years)	79.7 ± 5.2	78.5 ± 5.0	**0.032**
Male Sex, n (%)	92 (59.7)	88 (57.1)	0.644
Logistic EuroSCORE (%)	14.0 ± 2.7	13.7 ± 2.7	0.343
EuroSCORE II (%)	6.0 ± 3.0	6.2 ± 2.9	0.557
STS-PROM (%)	4.5 ± 2.2	4.2 ± 2.1	0.394
BMI (kg/m^2^)	27.1 ± 5.0	27.7 ± 4.7	0.270
Obesity, n (%)	36 (23.4)	40 (26.0)	0.574
Diabetes mellitus, n (%)	48 (31.2)	51 (33.1)	0.976
- dietary, n (%)	6 (3.9)	6 (3.9)	
- oral antidiabetics, n (%)	20 (13.0)	23 14.9)	
- insulin-dependent, n (%)	22 (14.3)	22 (14.3)	
Hypertension, n (%)	143 (92.9)	141 (91.6)	0.671
Hyperlipidaemia, n (%)	132 (85.7)	126 (81.8)	0.354
Nicotine abuse, n (%)	46 (29.9)	45 (29.2)	0.755
- history of smoking, n (%)	32 (20.8)	28 (18.2)	
- current smoking, n (%)	14 (9.1)	17 (11.0)	
Dialysis, n (%)	7 (4.5)	2 (1.3)	0.091
COPD, n (%)	28 (18.2)	22 (14.3)	0.354

Categorial variables are presented as number and percentegas, continuous variables as mean ± standard deviation. *STS – PROM* Society of thoracic surgeons – predicted risk of operative mortality, *BMI* body mass index, *COPD* chronic obstructive pulmonary disease, *TAVI* transcatheter aortic valve implantation. *PCI* percutaneous coronary intervention. *SAVR* surgical aortic valve replacement, *CABG* coronary artery bypass grafting

**Table 6:** Cardiovascular status in the matched cohort

	TAVI + PCI (*n* = 154)	SAVR + CABG (*n* = 154)	*p* value
Atrial fibrillation, n (%)	53 (34.4)	48 (31.2)	0.544
NYHA IV, n (%)	12 (7.8)	10 (6.5)	0.658
Extracardial atheropathy, n (%)	58 (37.7)	54 (35.1)	0.636
CVD, n (%)	41 (26.6)	37 (24.0)	0.600
PAD, n (%)	29 (18.8)	26 (16.9)	0.655
Pulmonary hypertension, n (%)	59 (38.3)	44 (28.6)	0.070
Previous stroke, n (%)	19 (12.3)	13 (8.4)	0.263
Previous myocardial infarction, n (%)	37 (24.0)	36 (23.4)	0.893
Previous cardiac surgery, n (%)	12 (7.8)	8 (5.2)	0.355
LVEF (%)	51.5 ± 11.0	50.9 ± 13.2	0.643
Aortic valve area (cm^2^)	0.78 ± 0.16	0.79 ± 0.25	0.638
Peak pressure gradient (mmHg)	64.3 ± 21.9	61.6 ± 23.5	0.380
Mean pressure gradient (mmHg)	40.5 ± 14.6	37.7 ± 17.0	0.170
Coronary artery disease, n (%)	154 (100.0)	154 (100.0)	1.000
- 1-CAD, n (%)	38 (24.7)	28 (18.2)	0.165
- 2-CAD, n (%)	53 (34.4)	47 (30.5)	0.465
- 3-CAD, n (%)	62 (40.3)	79 (51.3)	0.052
- main stem stenosis	32 (20.8)	45 (29.2)	0.087
SYNTAX Score I (%)	16.2 ± 10.9	22.2 ± 13.5	** < 0.001**
- low SYNTAX Score I (≤ 22), n (%)	97 (63.0)	73 (47.4)	**0.003**
- intermediate SYNTAX Score I (23–32), n (%)	20 (13.0)	27 (17.5)	0.287
- high SYNTAX Score I (> 32), n (%)	27 (17.5)	46 (29.9)	**0.012**

Categorial variables are presented as number and percentegas, continuous variables as mean ± standard deviation. *NYHA* New York Heart Association, *CVD* cerebrovascular disease, *PAD* peripheral arterial disease, *LVEF* left-ventricular ejection fraction, *CAD* coronary artery disease, *TAVI* transcatheter aortic valve implantation. *PCI* percutaneous coronary intervention, *SAVR* surgical aortic valve replacement. CABG: coronary artery bypass grafting

**Table 7:** Procedural characteristics in the matched cohort

	TAVI + PCI (*n* = 154)	SAVR + CABG (*n* = 154)	*p* value
Analgosedation, n (%)	61 (39.6)	0	** < 0.001**
Local anesthesia, n (%)	18 (11.7)	0	** < 0.001**
General anesthesia, n (%)	75 (48.7)	154 (100.0)	** < 0.001**
Procedural time (min)	107.0 (76.0–127.0)	265.0 (225.0–328.0)	** < 0.001**
Days between PCI and TAVI	29.5 (7.0–43.3)		

Categorial variables are presented as number and percentegas, continuous variables as mean ± standard deviation. *TAVI* transcatheter aortic valve implantation, *PCI* percutaneous coronary intervention, *SAVR* surgical aortic valve replacement, *CABG* coronary artery bypass grafting.

**Table 8:** Clinical outcomes in the matched cohort

	TAVI + PCI (*n* = 154)	SAVR + CABG (*n* = 154)	*p* value
Delirium, n (%)	10 (6.5)	48 (31.2)	** < 0.001**
Stroke, n (%)	3 (1.9)	12 (7.8)	**0.017**
Myocardial infarction, n (%)	4 (2.6)	2 (1.3)	0.410
Need for transfusions, n (%)	46 (29.9)	122 (79.2)	** < 0.001**
Major bleedings, n (%)	16 (10.4)	56 (36.4)	** < 0.001**
Major vascular complications, n (%)	6 (3.9)	7 (4.5)	0.777
Minor vascular complications, n (%)	15 (9.7)	12 (7.8)	0.546
Acute kidney injury, n (%)	19 (12.3)	45 (29.2)	**0.003**
- Grade 1	10 (6.5)	24 (15.6)	
- Grade 2	4 (2.6)	7 (4.5)	
- Grade 3	5 (3.2)	14 (9.1)	
Pacemaker implantation, n (%)	17 (11.0)	11 (7.1)	0.234
30-day mortality (%)	6 (3.9)	13 (8.5)	0.097
180-day mortality (%)	19 (12.4)	19 (12.5)	0.965
365-day mortality (%)	26 (17.0)	24 (15.9)	0.796
730-day mortality (%)	35 (23.3)	30 (20.4)	0.542
1095-day mortality (%)	48 (33.8)	38 (25.9)	0.139

Categorial variables are presented as number and percentegas, continuous variables as mean ± standard deviation. *TAVI* transcatheter aortic valve implantation, *PCI* percutaneous coronary intervention, *SAVR* surgical aortic valve replacement, CABG coronary artery bypass grafting

## Discussion

In this study, we compared two strategies of treating high-grade symptomatic aortic stenosis in combination with coronary artery disease. The interventional treatment followed a staged procedure protocol with treatment of the coronary artery disease first and the TAVI procedure following, whereas in case of surgery, a one-procedure strategy was followed.

Interestingly, despite higher initial bleeding and stroke incidences in the SAVR + CABG group, early mortality was not significantly different, although there was a trend towards lower mortality after TAVI and PCI at 30 days. However, later timepoints indicated a higher mortality in the TAVI + PCI group, which became a significant effect at three years after aortic valve replacement in the unmatched population. In the propensity score matched cohorts that were adequately balanced with regard to age, risk scores and comorbidities, this effect lost its statistical significance.

Several other studies have investigated the strategy of treating AS and CAD in elderly patients. Most recently, Khedi et al. have published the 12-month results from a multicentre, prospective, non-inferiority, randomised controlled trial comparing FFR-guided PCI plus TAVI versus SAVR plus CABG. They applied a complex primary endpoint consisting of all-cause mortality, myocardial infarction, disabling stroke, clinically driven target-vessel revascularisation, valve reintervention, and life-threatening or disabling bleeding at twelve months after treatment. The trial was positive meeting the non-inferiority criteria with signs for superiority for the FFR-PCI/TAVI group [16]. However, in addition to major technical differences (such as the randomised design and use of FFR-guided PCI) the trial also differs from our data in having a smaller patient population and a shorter follow-up period.

If only one of the two pathologies is singled out, randomised trials or their meta-analyses already indicate trends: In the SYNTAX trial, CAD was treated by either CABG or Taxus stents (first generation drug eluting stents). Though this stent platform has been outperformed meanwhile [17], its use allowed for non-inferior outcome of mortality in low- or mid-complex CAD patients (SYNTAX Score ≤ 32), whereas higher complexities (SYNTAX Score > 33), in particular three-vessel disease, displayed an increased rate of major adverse cardiac and cerebrovascular events (MACCE) after five years [18]. This finding was validated by a meta-analysis of 11 trials comprising 11.518 patients with multivessel disease, which showed a five-year all-cause mortality of 11.5% after PCI vs. 8.9% after CABG. The mortality difference was especially significant among diabetic patients (15.5% vs. 10.0%), but was not observed in non-diabetic patients or those with left main disease [19]. Moreover, a large meta-analysis of TAVI and SAVR in seven randomised controlled trials across high, intermediate and low risk levels (PARTNER 1 A, 2 A, 3, CoreValve US, NOTION, SURTAVI) provided follow-up for more than three years and revealed that an initial survival advantage of TAVI vs. SAVR in the first six months was lost and reversed to favouring SAVR over TAVI after 24 months [20].

These results suggest that a combination of both entities could replicate an early advantage of the interventional strategy, but also a long-term disadvantage, at least in certain patient groups. Several other reports have been probing the competing strategies of either complete interventional or complete surgical or hybrid treatments. In 2018, Barbanti and colleagues reported results from the Italian OBSERVANT multicentre cohort study. In 472 propensity score matched patients for either strategy, the TAVI + PCI group (with 92% of PCIs done before and 8% at the time of TAVI) did not statistically differ from the SAVR + CABG cohort, although there were 25% fewer deaths in the surgical group after three years (59 vs. 79). Notably, the observed mortality rates at the 3-year timepoint were very similar to our results with 33.5% in the interventional and 25.0% in the surgical group [21].

In 2019, Søndergaard and colleagues used the prospective SURTAVI data set of 1660 intermediate-risk AS patients and analysed 332 cases of concomitant coronary revascularisation for two years. The distribution pattern of CAD was mild (including a SYNTAX Score mean of 8.3%), with > 60% of cases presenting as 1-vessel treatment and < 10% in each group requiring 3-vessel approaches. As a result, the 169 interventionally treated patients were statistically indistinguishable from the 163 surgically treated patients with mortality rates of 16% vs. 14% at two-year follow up (p = 0.62) [22].

Following up, in 2021, Alperi and colleagues included 156 pairs of propensity score matched patients with complex coronary artery disease (SYNTAX Score of 26.3 vs. 26.9 and left main involvement of 55.8% vs. 57.1% in the interventional vs. surgical group, respectively) in their analysis. Again, no statistical difference was found in mortality at five years between interventional (38.1%) and surgical patients (32.0%), though the follow-up was unevenly distributed, with the interventional cases at five years too rare for statistical response [23].

In contrast, Baumbach and colleagues reported a prospective single centre cohort study of 626 patients with either surgical (SAVR + CABG, 464 patients), interventional (TAVI + PCI, 112 patients) or hybrid treatment (TAVI + OP/MIDCAB, 50 patients). Three-year follow-up revealed a significant survival adavantage in the surgical group (80% vs. 49%, p < 0.001). Of note, the mortality in the hybrid group resided exactly with the purely interventional group. However, the potential impact of this study’s finding was limited by age, EuroSCORE I and left ventricular function all significantly favoring the surgical group, in addition to a lack of propensity score matching and a follow-up adherence of 53% for the surgical cohort or even lower for the other groups [24]. Nevertheless, this study is particularly noteworthy as it provides the first significant evidence suggesting a potential long-term advantage of surgical treatment for AS and CAD. In our study, even with propensity score matching and a follow-up adherence of 98.1% for one year and 90.9% for three years, the surgical group was trending towards a survival advantage until the end of the three-year observation period.

Moreover, Amat-Santos and coworkers published a propensity score matched retrospective study of the Spanish TAVI registry in 2024, revealing significantly higher rates of the combined primary endpoint of stroke and mortality in the surgical group after 30 days and one year (15.8% vs. 9.9%, p = 0.033) in a population at low risk (STS-PROM of 3.0% and 3.4% after matching) [25]. Of note, the mortality at one year was similar in both groups (10.1 vs. 7.1%, p = 0.291), bearing similarities to our study in an intermediate-risk cohort.

Further research is warranted beyond the existing prospective and retrospective data to optimise treatment strategies for intermediate-risk patients presenting with AS and CAD.

### Study limitations

There are limitations to this study. Firstly, because of its retrospective design, acquiring certain types of data, e.g. causes of death, was severely limited, resulting in the fact that cardiovascular mortality in particular could not be reported for the entire study population. A subgroup analysis on cardiovascular mortality is included in the supplementary data under 4). Secondly, data on the SYNTAX Score was missing for 16 patients in the TAVI + PCI group and 8 patients in the SAVR + CABG group, which is why this variable could not be included in the propensity score matching and remained significantly different between the two treatment groups. In addition, there are potentially important factors for clinical outcome that were not examined in this study, including complete revascularisation and prothesis degeneration. Subgroup analyses for coronary and valve reinterventions, however, can be found in the supplementary data under 5). Thirdly, the EuroSCOREs that were used as an inclusion criterion in this study are now outdated and surgical risk scores in general are not entirely suitable for TAVI patients. Lastly, interventions that date back to 2012 were included in this study and the techniques and materials used in percutaneous procedures have significantly improved since then.

## Conclusion

To conclude, we report that an intermediate-risk patient population presenting with aortic stenosis and concomitant coronary artery disease derives long-term benefit from both TAVI + PCI as well as SAVR + CABG strategies. At three years, and only then, the unmatched and younger surgical cohort seemed to benefit more from the SAVR + CABG procedure, although the CAD-pattern was more complex in these cases. Overall, for elderly intermediate-risk AS patients with complex CAD, surgery remains a viable option; however, the increased risk of stroke must be carefully considered.

## Supplementary Information

Below is the link to the electronic supplementary material.

ESM 1 (DOCX 25.2 KB)

## Data Availability

The data that support the findings of this study are available from the corresponding author upon reasonable request.
