# Supplementary material for: TAVI plus PCI versus SAVR plus CABG: Long-term outcome of a multicentre-registry
Source: Clin Res Cardiol. 2025 Sep 11;115(7):1188–98. doi: 10.1007/s00392-025-02755-9 (PMC13249651; doi:10.1007/s00392-025-02755-9)
Supplement: Supplementary file 1 — (DOCX 25.2 KB) [file 392_2025_2755_MOESM1_ESM.docx]

Supplements

1. Detailed study population

- total: 366 patients (211 TAVI+PCI, 155 SAVR+CABG)
- Munich: 73 patients (43 TAVI+PCI, 30 SAVR+CABG), treated between 2015 and 2019
- Kiel: 163 patients (99 TAVI+PCI, 64 SAVR+CABG), treated between 2012 and 2020
- Bad Oeynhausen: 130 patients (69 TAVI+PCI, 61 SAVR+CABG), treated between 2016 and 2019

1. Detailed follow-up data

|  | TAVI + PCI (n=211) | SAVR + CABG (n=155) | p value |
| --- | --- | --- | --- |
| 30-day mortality (%)   - missing | 10 (4.8)  1 | 13 (8.4)  1 | 0.159 |
| 180-day mortality (%)   - missing | 26 (12.4)  1 | 19 (12.4)  3 | 0.991 |
| 365-day mortality (%)   - missing | 38 (18.1)  1 | 24 (15.7)  3 | 0.547 |
| 730-day mortality (%)   - missing | 51 (24.9)  6 | 30 (20.1)  7 | 0.294 |
| 1095-day mortality (%)   - missing | 72 (37.1)  17 | 38 (25.5)  7 | **0.022** |

Unmatched population:

|  | TAVI + PCI (n=154) | SAVR + CABG (n=154) | p value |
| --- | --- | --- | --- |
| 30-day mortality (%)   - missing | 6 (3.9)  1 | 13 (8.5)  1 | 0.097 |
| 180-day mortality (%)   - missing | 19 (12.4)  1 | 19 (12.5)  2 | 0.965 |
| 365-day mortality (%)   - missing | 26 (17.0)  1 | 24 (15.9)  3 | 0.796 |
| 730-day mortality (%)   - missing | 35 (23.3)  4 | 30 (20.4)  7 | 0.542 |
| 1095-day mortality (%)   - missing | 48 (33.8)  12 | 38 (25.9)  7 | 0.139 |

Matched population:

1. NYHA class progression (TUM Klinikum, HDZ NRW, Heart Surgery UKSH Kiel)

|  | TAVI + PCI (n=112) | SAVR + CABG (n=155) | p value |
| --- | --- | --- | --- |
| NYHA at baseline |  |  | 0.332 |
| - I, n (%) | 4 (3.6) | 6 (3.9) |  |
| - II, n (%) | 37 (33.0) | 68 (43.9) |  |
| - III, n (%) | 63 (56.3) | 71 (45.8) |  |
| - IV, n (%) | 8 (7.1) | 10 (6.5) |  |
| NYHA at follow-up |  |  | 0.059 |
| - I, n (%) | 29 (25.9) | 6 (3.9) |  |
| - II, n (%) | 23 (20.5) | 5 (3.2) |  |
| - III, n (%) | 16 (14.3) | 5 (3.2) |  |
| - IV, n (%) | 1 (0.9) | 3 (1.9) |  |
| - missing | 43 | 136 |  |

1. Cardiovascular mortality (Subgroup HDZ NRW)

|  | TAVI + PCI (n=69) | SAVR + CABG (n=61) | p value |
| --- | --- | --- | --- |
| Cardiovascular mortality, n (%) | 1 (1.4) | 5 (8.2) | 0.060 |
| - missing | 13 | 13 |  |

1. Reintervention

Coronary reintervention (all centres)

|  | TAVI + PCI (n=211) | SAVR + CABG (n=155) | p value |
| --- | --- | --- | --- |
| Coronary reintervention, n (%) | 19 (9.0) | 3 (1.9) | **<0.001** |
| - missing | 68 | 27 |  |

Valve reintervention (HDZ NRW, Heart Surgery UKSH Kiel, DHZ Munich)

|  | TAVI + PCI (n=69) | SAVR + CABG (n=155) | p value |
| --- | --- | --- | --- |
| Valve reintervention, n (%) | 1 (1.4) | 5 (3.2) | 0.334 |
| - missing | 0 | 28 |  |
